# Genetic basis and selection of glyceollin elicitation in wild soybean

**DOI:** 10.3389/fpls.2024.1240981

**Published:** 2024-02-28

**Authors:** Farida Yasmin, Hengyou Zhang, Larry Leamy, Baosheng Wang, Jason Winnike, Robert W. Reid, Cory R. Brouwer, Bao-Hua Song

**Affiliations:** ^1^ Department of Biological Sciences, The University of North Carolina at Charlotte, Charlotte, NC, United States; ^2^ Key Laboratory of Plant Resources Conservation and Sustainable Utilization, South China Botanical Garden, Chinese Academy of Sciences, Guangzhou, China; ^3^ Center of Conservation Biology, Core Botanical Gardens, Chinese Academy of Sciences, Guangzhou, China; ^4^ David H. Murdock Research Institute, Kannapolis, NC, United States; ^5^ Department of Bioinformatics and Genomics, The University of North Carolina at Charlotte, Charlotte, NC, United States

**Keywords:** epistasis, mGWAS, phytoalexin, candidate gene, gene cluster, *Glycine soja*

## Abstract

Glyceollins, a family of phytoalexins elicited in legume species, play crucial roles in environmental stress response (e.g., defending against pathogens) and human health. However, little is known about the genetic basis of glyceollin elicitation. In the present study, we employed a metabolite-based genome-wide association (mGWA) approach to identify candidate genes involved in glyceollin elicitation in genetically diverse and understudied wild soybeans subjected to soybean cyst nematode. In total, eight SNPs on chromosomes 3, 9, 13, 15, and 20 showed significant associations with glyceollin elicitation. Six genes fell into two gene clusters that encode glycosyltransferases in the phenylpropanoid pathway and were physically close to one of the significant SNPs (ss715603454) on chromosome 9. Additionally, transcription factors (TFs) genes such as *MYB* and *WRKY* were also found as promising candidate genes within close linkage to significant SNPs on chromosome 9. Notably, four significant SNPs on chromosome 9 show epistasis and a strong signal for selection. The findings describe the genetic foundation of glyceollin biosynthesis in wild soybeans; the identified genes are predicted to play a significant role in glyceollin elicitation regulation in wild soybeans. Additionally, how the epistatic interactions and selection influence glyceollin variation in natural populations deserves further investigation to elucidate the molecular mechanism of glyceollin biosynthesis.

## 1 Introduction

Plants synthesize a wide array of specialized metabolites, also referred to as secondary metabolites or phytochemicals. These compounds play crucial roles in facilitating plant adaptation to dynamic environments, ensuring survival, and presenting potential applications for human use (Ahmed and Kovinich, 2021). Phytoalexins are specialized metabolites synthesized *de novo* in response to various biotic and abiotic stresses. Examples include indole alkaloid camalexin in *Arabidopsis*, phenolic aldehyde gossypol in cotton, phenylpropanoid stilbenes in grapevines, isoflavonoid-derived glyceollins in legume, and momilactones and phytocassanes terpenoids in rice (Jeandet et al., 2002; Wang et al., 2009; Donnez et al., 2011; Saga et al., 2012; Yamamura et al., 2015; Jahan et al., 2019; Jeandet et al., 2020). Among these phytoalexins, isoflavonoids have been of research interest due to the various pharmacological properties and essential roles in plant defense (Dixon and Steele, 1999). The major isoflavones identified in soybeans are comprised of genistein, daidzein, and glycitein (Murphy et al., 2002). It has been reported that trace amounts of glyceollins are produced transiently from daidzein under the influence of both abiotic and biotic stresses. This observation suggests that the production of glyceollins, to a significant extent, is contingent upon external stress factors (Subramanian et al., 2006; Aisyah et al., 2013; Lygin et al., 2013; Bamji and Corbitt, 2017; Jahan and Kovinich, 2019; Jahan et al., 2019). In this regard, producing glyceollins contributes to multiple beneficial effects, such as fostering symbiosis between soybean and *Bradyrhizobium japonicum* and inhibiting the growth of various microbes (Graham and Graham, 1996; Subramanian et al., 2006). Moreover, they have properties that are beneficial to human health, such as anti-cancer, antioxidant, and neuroprotective (Kim et al., 2012; Nwachukwu et al., 2013; Bamji & Corbitt, 2017; Seo et al., 2018; Pham et al., 2019). However, studies on glyceollins are mainly focused on their medicinal properties, and to the best of our knowledge, little is known about how their elicitation is regulated.

To date, few genes have been identified associated with glyceollin biosynthesis. For example, two key transcription factors, known as *GmNAC42-1* and *GmMYB29A2*, were identified play a crucial role in the biosynthesis of glyceollin I in soybeans, and they contribute to resistance against *Phytophthora sojae* (Jahan et al., 2019; Jahan et al., 2020). In a study conducted by Jahan and colleagues in 2019, acidity stress was employed to elicit the biosynthesis of glyceollin. They observed that the overexpression of *GmNAC42-1* in hairy roots resulted in a remarkable increase of over 10-fold in glyceollin production. The NAC-family transcription factor *GmNAC42-1* plays a crucial role in regulating certain glyceollin biosynthesis genes, though not all. This suggests that there is still unidentified essential transcription factor(s) within the glyceollin gene regulatory network (Jahan et al., 2019). In a separate investigation conducted by Jahan and colleagues in 2020, it was revealed that upon stimulation with wall glucan from *P. sojae*, *GmMYB29A2* interacted with the promoters of two glyceollin I biosynthesis genes *in vitro* and *in vivo*. This interaction led to the accumulation of glyceollin I and the expression of resistance against *Phytophthora* (Jahan et al., 2020). Given that glyceollins are produced in trace amounts and transiently under stress conditions, finely adjusting these transcription factors emerges as a promising strategy to enhance their production efficiently.

Phytoalexins have been considered the target of natural selection due to their activities in biotic and abiotic stress responses in natural environments (Pichersky and Gang, 2000; Qi et al., 2004; Miyamoto et al., 2016). Research has shown that genomic approaches in crop wild relatives can reveal genes responsible for target metabolites (Zhang et al., 2017b). Improvements can be achieved by manipulating the metabolic pathway in crops. Examples of this phenomenon include 7-epizingiberene synthase *(ShZIS)*, a sesquiterpene synthase specific to trichomes that is involved in the naturally optimized sesquiterpene biosynthetic pathway in wild tomatoes. This enzyme enhances cultivated tomato resistance against various herbivores when subjected to genetic engineering (Bleeker et al., 2012). Mipeshwaree Devi et al. (2023) have comprehensively summarized recent advancements in the realm of metabolic engineering, specifically focusing on plant-specialized metabolites. Notably, Zhang and colleagues (2022) employed CRISPR/Cas9 for targeted mutagenesis in *GmUGT*, a UDP-glycosyltransferase pivotal in flavonoid biosynthesis. This targeted mutagenesis resulted in enhanced resistance against leaf-chewing insects (Zhang et al., 2022). Therefore, understanding the metabolic pathways and their regulatory mechanisms is essential for targeted metabolite engineering to achieve crop improvement. However, there is limited reported progress in the field of metabolic engineering (Mipeshwaree Devi et al., 2023).

Furthermore, the study of metabolic gene clusters, which are groups of co-localized and potentially coregulated non-homologous genes involved in specific metabolic pathways, has gained attention (Nützmann et al., 2016; Töpfer et al., 2017). While these clusters have long been observed in microbial genetics, their existence in plant metabolic pathways has only recently been explored (Zheng et al., 2002; Rocha, 2008; Koonin, 2009). A study by Chae et al. (2014) focusing on metabolic gene clusters in *Arabidopsis*, soybean, sorghum, and rice suggested that approximately one-third of all the metabolic genes in *Arabidopsis*, soybean, and sorghum, and one-fifth in rice were rich in gene clusters across primary and specialized metabolic pathways (Chae et al., 2014). There is compelling evidence indicating that the highly plastic plant genome itself generates metabolic gene clusters via gene duplication, neofunctionalization, divergence, and genome reorganization instead of horizontal gene transfer from microbes (Osbourn and Field, 2009). This suggests that plants rewire their genome to gain new adaptive functions driven by the need to survive in distinct environments. Mining and functional validation of the candidate genes in such clusters will facilitate the discovery of new enzymes and chemistries that render pathway prediction. Moreover, metabolic gene clusters are likely to be located within dynamic chromosomal regions, and thus, many identified so far may be due to recent evolution (Qi et al., 2004; Field et al., 2011; Matsuba et al., 2013). If so, investigation of these clusters can provide insights into their evolutionary history. The vast and diverse array of specialized metabolites produced through multi-step metabolic pathways plays an essential role in plant adaptation to various ecological niches. However, the occurrence, prevalence, and evolution of such gene clusters in plants are largely unknown. Thus, the study of plant metabolic gene clusters has implications for molecular biology and evolutionary genomics (Yeaman and Whitlock, 2011; Takos and Rook, 2012; Nützmann et al., 2016; Chavali and Rhee, 2018).

To the best of our knowledge, due to the extraordinary metabolic diversity, less than 50 plant-specialized metabolic pathways have been biochemically and genetically identified to date (Nützmann et al., 2016). Metabolomic GWAS (mGWAS) offers an effective approach to understanding the genetic basis of metabolites and their associated traits (Chan et al., 2010; Chan et al., 2011; Riedelsheimer et al., 2012; Luo, 2015). mGWAS allows the identification of common polymorphic regions controlling complex metabolic traits by substantially increasing association panel and genome-wide molecular markers. Besides elucidating genetic architecture, mGWAS can also be used to infer gene functions (Luo, 2015). Hence, mGWAS provides a comprehensive approach to discovering candidate genes. Thus far, it has been used to uncover the genetic basis of variations of a number of different metabolites. For example, Chen et al. (2014) carried out a rice mGWAS study that identified 36 candidate genes influencing the variation of metabolites with physiological and nutritional importance (Chen et al., 2014). Additionally, Petersen et al. (2012) illustrated that in an association study (i.e., mGWAS), a ratio between two metabolite concentrations provides more insightful information than the concentrations of the two metabolites individually. Implementing this innovative approach in mGWAS proves to be valuable for revealing novel and biologically significant associations. They emphasized several studies in which the incorporation of metabolite ratios in both genome-wide and metabolite-wide association studies significantly strengthened the associations (Petersen et al., 2012). For instance, Gieger et al. (2008); Illig et al. (2010), and Suhre et al. (2011) illustrated that the utilization of metabolite ratios in GWAS studies resulted in a substantial increase in the power of association, reaching tens of orders of magnitude (Gieger et al., 2008; Illig et al., 2010; Suhre et al., 2011).

The isoflavonoid pathway has been relatively well studied (Yoneyama et al., 2016; Sukumaran et al., 2018). However, a gap in our understanding of the genetic basis of glyceollin elicitation remains. As of now, researchers have identified transcription factors crucial for the regulation of glyceollin biosynthesis, such as *GmNAC42-1* and *GmMYB29A2* (Jahan et al., 2019; Jahan et al., 2020). In the present study, we selected wild soybean (*Glycine soja*), a wild relative of soybean (*Glycine max)*, to delineate the genetic basis and evolution of glyceollin accumulation resulting from biotic stress, i.e., soybean cyst nematode (SCN), the most devastating soybean pest worldwide (Tylka and Marett, 2021). Wild soybeans thrive in diverse habitats and harbor much higher, underexplored genetic diversity than cultivated soybeans (Zhang et al., 2019). Hence, it is an ideal system to understand the genetic basis and evolution of glyceollin variation. Eventually, the essential genes identified in wild soybeans can be used for metabolic engineering or in a breeding program to develop nutrition-rich biofortified soybean cultivars as they exhibit similar genome size and content with no reproductive barriers (Singh and Hymowitz, 1999). In this study, we aim to address these three questions: (1) What is the genetic basis of variation in glyceollin elicitation by SCN? (2) Are there any gene clusters and transcription factors involved in glyceollin variation? (3) Are epistatic interactions and natural selection important evolutionary factors influencing the variation of glyceollin elicitation in natural populations? Our study is the first to employ genomic and evolutionary approaches to understand the genetic basis and selection of glyceollin elicitation. The results provide a fundamental basis for the long-term goal of developing glyceollin-fortified soybean cultivars.

## 2 Materials and methods

### 2.1 Plant materials

A total of 265 accessions of wild soybean, *Glycine soja*, from a wide geographic range, originally collected from China, Japan, Russia, and South Korea, were utilized (**Supplementary Table 1**). The seeds of these ecotypes were obtained from the USDA National Germplasm resources laboratory (https://www.ars-grin.gov/).

### 2.2 Plant preparation, SCN inoculation, and sample collection

Seed preparation, germination, transplanting, and soybean cyst nematode (SCN, *Heterodera glycines Ichinohe*, HG type 1.2.5.7) inoculation were performed following a previously developed protocol (Zhang and Song, 2017; Zhang et al., 2017a). Specifically, each wild soybean ecotype seed underwent surface sterilization using a 0.5% sodium hypochlorite solution for one minute, followed by thorough rinsing. These sterilized seeds were then germinated on sterile filter paper in petri dishes containing an appropriate amount of sterile water for a duration of 3 to 4 d. Once germinated, it was transplanted into a cone-tainer (Greenhouse Megastore, Danville, IL, USA), utilizing sterile sand as the growth medium. The arrangement of cone-tainers in a cone-tainer tray (Greenhouse Megastore, Danville, IL, USA) followed a randomized complete block design. To ensure optimal growth conditions, all the plants were kept within a growth chamber maintained at a temperature of 27°C, with a relative humidity of 50%, and subjected to a long-day photoperiod of 16 h of light followed by 8 h of darkness. The seedlings received regular daily watering to maintain adequate moisture levels for healthy growth.

For SCN inoculation, the HG type 1.2.5.7 nematodes stocks were maintained in a controlled greenhouse environment, with a consistent temperature of 27°C and a photoperiod of 16 h of light followed by 8 hof darkness, spanning over 30 generations. To isolate female nematodes, they were carefully extracted from the roots of soybean cv. Hutcheson by gently massaging the roots in water and then filtering the solution through nested sieves with mesh sizes of 850 and 250 micrometers. The collected female nematodes were then crushed using a rubber stopper in an 8-inch diameter sieve with a 250-micrometer mesh, releasing the eggs, which were subsequently collected using a 25-micrometer mesh sieve. For further purification, the eggs underwent a modified sucrose flotation method (Matthews et al., 2003).

Following purification, the eggs were placed on moist paper tissues and placed in a plastic tray filled with 1 centimeter of water. The tray was covered with aluminum foil and maintained at a temperature of 27°C. Three days after hatching, the second-stage juvenile nematodes (referred to as J2) were harvested and concentrated to achieve a final concentration of 1,800 J2 per milliliter in a 0.09% agarose suspension. After three days of transplantation, when the seedlings were healthy and displayed uniform growth, they were inoculated with 1 milliliter of the J2 nematode inoculum. Concurrently, seedlings inoculated with a 0.09% agarose solution served as the control group.

Whole root tissues were collected and weighed five days post-infection (dpi). The 5 dpi time point was chosen because our previous study suggested a significant inhibition in SCN development in a resistant genotype compared to normal growth in a susceptible genotype (Zhang et al., 2017a). All samples were flash-frozen in liquid nitrogen and stored at -80°C. Four biological replicates per wild soybean genotype were used, eventually a total of 1,020 samples.

### 2.3 Metabolite extraction and quantification

We employed the extraction method of metabolites from root tissue described in Strauch et al. (2015) (Strauch et al., 2015). The soybean root samples underwent homogenization within a ball mill homogenizer, utilizing an extraction solvent that featured daidzein-d6 (Biotek, catalog#BT-387818) as an internal standard. The metabolite profiling was provided by the service from David H. Murdock Research Institute at the North Carolina Research Campus employing UPLC-MS/MS (ultraperformance liquid chromatography-tandem mass spectrometry). Method development and analysis were conducted using a Waters ACQUITY UPLC-Quattro Premier XE MS. The UPLC and MS/MS parameters were established through experimentation with test samples and analytical standards of glyceollin (chemically synthesized by Dr. P. Erhardt at University of Toledo), daidzein (Sigma Aldrich, catalog#D7802), and daidzein-d6 (LGCstandards). The MS/MS acquisition parameters were optimized based on the analytical standards. Additionally, optimized UPLC gradient conditions were determined to effectively separate the glyceollin and daidzein peaks. Peaks that were consistently detected in at least three biological replicates within each genotype were used for downstream analyses. Each metabolite was confirmed using pure standard compounds, including daidzein, daidzein-d6, and glyceollin. Due to the low concentrations of these compounds and the small sample masses of the wild soybean root samples that had been collected, we used a signal-to-noise ratio of ≥10 for the measurement of the peaks for glyceollin and daidzein. Our method successfully measured daidzein (μg/g root) and glyceollin (unitless) in 264 accessions of wild soybean *G. soja* roots quantitatively and semi-quantitatively, respectively. Following method development, optimization, and analyses of the test samples, calibration curves were designed using at least six different concentrations of daidzein, created in triplicate to quantify known concentrations of daidzein and glyceollin. A second-degree polynomial was derived from the known concentrations of the standard curve samples and the mass spectrometer response (daidzein/internal standard) from the standard curve data. The resulting polynomial was used to calculate the concentrations of daidzein in the experimental samples. Low, medium, and high QC (quality control) samples were created to assess the accuracy of the calculations. We used the ratio of glyceollin (unitless, a semi-quantitative measurement of glyceollin) to daidzein (μg/g root) (GVSD) as our phenotypic trait (**Supplementary Table 1** and **Supplementary Figure 2**). This phenotype henceforth is denoted GVSD. The justification for employing the ratio is to enhance statistical power by minimizing variability in the metabolomic data and mitigating experimental errors associated with data noise (Petersen et al., 2012).

### 2.4 Genotypic data

Genotype data for the 264 accessions were obtained from SoySNP50K (Song et al., 2013), which was downloaded from SoyBase (SoyBase.org). After the filter, the genotype included 32,976 genome-wide single nucleotide polymorphic markers (SNPs) with a minor allele frequency (MAF) of at least 5% and a missingness rate of less than 10%.

### 2.5 Metabolite-based genome-wide association study and linkage disequilibrium estimation

Our genome-wide association analysis was conducted on GVSD (a ratio of glyceollin mean to daidzein mean) in response to SCN infection on all 264 ecotypes using the GAPIT R package (2.0) (Tang et al., 2016). To minimize false-positive associations, we controlled population structure among genotypes with four principal components as calculated with the GAPIT. Heritability estimate and SNP effect were calculated by running GWAS applying CMLM and MLM methods, respectively, implemented in the GAPIT R package (2.0) (Tang et al., 2016).

The Manhattan plot was generated using the R package *qqman* (Turner, 2018). In addition to the genome-wide significant threshold, we also calculated the chromosome-wide Bonferroni thresholds using independent SNPs estimated on each chromosome following the method of Li and Ji (2005) (Li and Ji, 2005). Linkage disequilibrium (LD) was calculated across the panel with the TASSEL program, version 5 (Bradbury et al., 2007), for the significant SNPs identified from the GWAS analysis. LD was measured using squared correlation R-squared (r^2^) of 0.2 (upper right in the LD plot) and *p*-value < 0.05 (the lower left in the LD plot). A pairwise LD was generated following the R function described by Shin et al. (2006) (Shin et al., 2006). Genes within LD blocks containing significant SNPs were identified as potential sources of candidates for further analyses.

### 2.6 Identification of candidate genes

For extensive gene mining, a pairwise linkage disequilibrium (LD) analysis was initially used for potential candidate gene identification. Then, genes in each LD block were examined as potential candidate genes, and their annotations were obtained from the Phytozome v13 database (Goodstein et al., 2011). Afterward, a GO enrichment analysis of the identified candidate genes was performed using ShinyGO v0.66: Gene Ontology Enrichment Analysis (*p*-value cutoff (FDR, false discovery rate) = 0.05) (Ge et al., 2020), SoyBase GO Enrichment Data (Grant et al., 2010). To investigate the involvement of these potential candidate genes in metabolic pathways, a database search was performed through an annotation file from Phytozome v13 (Goodstein et al., 2011), SoyBase (Grant et al., 2010), SoyCyc 10.0 Soybean Metabolic Pathway (Hawkins et al., 2021), and Pathview databases (Luo et al., 2017). Finally, a PMN plant metabolic cluster viewer was applied to categorize enzymes into classes (signature or tailoring) and metabolic domains (Hawkins et al., 2021).

### 2.7 Analysis of epistatic interactions

For any significant SNPs uncovered in the GWAS analysis, it is useful to test whether, beyond their direct effects, they also exhibited interactive effects on GVSD. To accomplish this, we first produced numerically formatted genotypes, in which the homozygous genotype index value is 1 and -1 and the heterozygous 0. This allows us to test for epistasis for each pairwise combination in a simple general linear model with 1 degree of freedom for the additive effects of each of the two SNPs and their interaction. We included the first four principal components from the GAPIT analysis in the model to be consistent with the GWAS scan, where these components were used to adjust for structural relatedness (see below). The significance of all interactions was evaluated with the sequential Bonferroni procedure. To illustrate the interactions of SNP pairs, we also calculated regressions of GVSD on each SNP, but at each of the three genotypes (using the -1, 0, and 1 index values) of the second SNP involved in the significant interaction.

### 2.8 Extended haplotype homozygosity analysis

To test allele-specific selection patterns of the identified significant SNPs, we analyzed extended haplotype homozygosity (EHH, (Sabeti et al., 2002)) for each significant SNP. The EHH analysis was conducted in SELSCAN v.1.2.0a (Szpiech and Hernandez, 2014) with default parameters, and only SNPs with MAF > 0.05 was used in this analysis.

## 3 Results

### 3.1 Genomic dissection of glyceollin accumulation upon biotic elicitation

**Figure 1:**
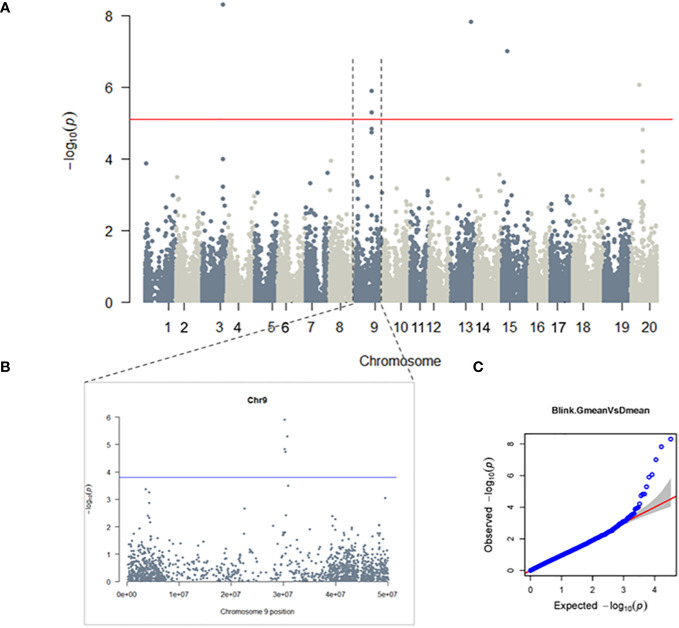
GWAS of Glyceollin elicitation with SCN stress: A genome-wide **(A)** and chromosome-wide **(B)** Manhattan plots, with thresholds of 5.104 and 3.803, respectively; **(C)** quantile-quantile (QQ) plot. Significant SNPs are found on chromosomes 3, 9, 13, 15 and 20 at a 5% genome-wide threshold, the probability of 7.86×10^-6^ resulted in a threshold of 5.01 (solid red line in the genome-wide Manhattan plot) **(A)**. The 5% chromosome-wide LOD threshold resulted in significant p-values of 1.57×10^-4^ (threshold 3.803, solid blue line) **(B)**.

To investigate the genetic basis of glyceollin elicitation, we performed a metabolite-based genome-wide association study (mGWAS) of glyceollin content in wild soybean roots infected with soybean cyst nematode (SCN). The mGWAS identified a total of eight significant SNPs, with four (ss715603454, ss715603455, ss715603462, and ss715603471) located on chromosome 9 and ss715585948, ss715615975, ss715620269 and ss715636844 on chromosomes 3, 13, 15, and 20, respectively (**Figure 1A**; **Table 1**). These significant SNPs were identified based on both genome-wide Bonferroni threshold of 5.104 and chromosome-wide Bonferroni thresholds that varied narrowly from 3.79 to 3.82 among the 20 chromosomes (3.803 on chromosome 9) (**Figures 1A, B**; **Supplementary Table 2**). The mGWAS are visualized with the Manhattan and Q-Q (quantile-quantile) plots as shown in **Figure 1**. The four significant SNPs ss715603454, ss715603455, ss715603462, and ss715603471 on chromosome 9 at positions 30262482, 30191235, 30393285, and 30725658, respectively, are located closely to each other within a 535-kb genomic region (**Supplementary Table 2**). The heritability for glyceollin was estimated at 35%, suggesting that glyceollin elicitation was genetically controlled (**Supplementary Table 2**).

**Table 1:** Identification of significant SNPs and functional annotation of the plausible candidate genes.

Significant SNP	Chromosome	Functional annotation of associated genes
ss715585948	Gm03	*WRKY* family transcription factor family protein Zinc fingers superfamily protein
ss715603454	Gm09	UDP-glucosyl transferase 88A1 *RING/U-box* superfamily protein, *RING/FYVE/PHD* zinc finger superfamily protein *WRKY* family transcription factor family protein *MYB* domain Zinc fingers superfamily protein *Cytochrome P450* enzyme family Zinc finger, *RING-type*; Transcription factor jumonji/aspartyl beta-hydroxylase
ss715603455	Gm09	
ss715603462	Gm09	
ss715603471	Gm09	
ss715615975	Gm13	*bZIP* transcription factor *RING/U-box* superfamily protein, *RING/FYVE/PHD* zinc finger superfamily protein Zinc fingers superfamily protein *NAC* transcription factors *Cytochrome P450* enzyme family
ss715620269	Gm15	*RING/U-box* superfamily protein, *RING/FYVE/PHD* zinc finger superfamily protein *WRKY* family transcription factor family protein *MYB* domain
ss715636844	Gm20	UDP-Glycosyltransferase superfamily protein UDP-glucosyl transferase 85A2 hydroxy methylglutaryl CoA reductase 1 *Cytochrome P450*, family 71, subfamily B, polypeptide 34 cytochrome p450 79a2 *RING/U-box* superfamily protein, *RING/FYVE/PHD* zinc finger superfamily protein Zinc fingers superfamily protein

### 3.2 Candidate gene identification

**Figure 2:**
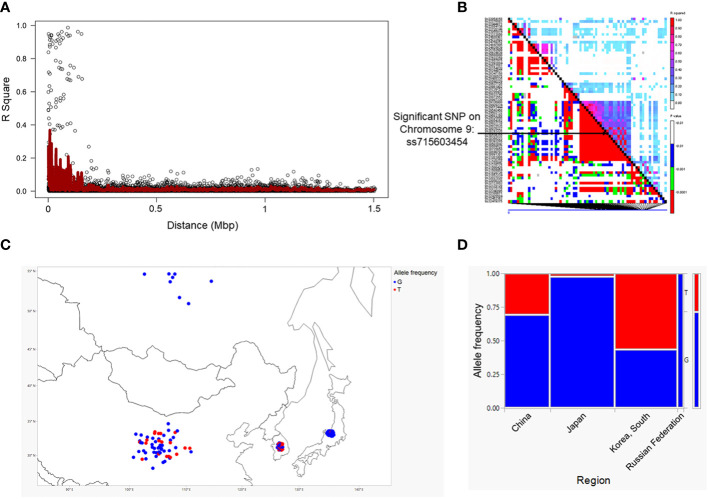
Linkage disequilibrium (LD) decay measured as R2 as a function of marker distance in the studied panel **(A)** and LD plot for chromosome 9 for significant SNPs. The black diagonal denotes LD between each site and itself **(B)**. Geographic range of the alleles of significant SNPs close to the gene clusters on chromosome 9 **(C)**. Allele frequency in each population. Allele frequency in different geographic regions for a significant SNP was generated using JMP^®^, Version *15*. SAS Institute Inc., Cary, NC, 1989–2021. **(D)**.

We employed a pairwise linkage disequilibrium (LD) analysis to identify potential candidate genes. For candidate gene determination, we considered *r2>0.2* as a cutoff for our LD analysis, where *r* ^2^ is the extent of allelic association between a pair of sites (Weir, 1990). **Figure 2A** shows the LD decay plot in the studied panel. We identified a total of 666 possible candidate genes within either side of 200 kb covering linkage disequilibrium (LD) blocks of the eight significant SNPs (soybean reference genome *Glycine max* Wm82.a2.v1) (Goodstein et al., 2011; Zhou et al., 2015). Further refining our selection, we narrowed the list to 51 candidate genes, focusing on the eight significant SNPs within the mentioned LD block region. Another criterion for this selection was the alignment with our pathway of interest, demonstrating a strong correlation with the target metabolites (**Supplementary Table 3**). The LD block within either side of the 200 kb region on chromosome 9 showed the strongest LD compared to the LD blocks for other significant SNPs identified on chromosomes 3, 13, 15 and 20 (**Figure 2B**; **Supplementary Figure 1**). Specifically, the candidate gene *Glyma.09G128200* exhibits the highest level of linkage disequilibrium (LD) near the significant SNP ss715603454 on chromosome 9 in comparison to the LD block associated with the remaining significant SNPs on this chromosome (**Figure 2B**). The functional annotation of the candidate genes on chromosome 9 (i.e., *Glyma.09G127700*, *Glyma.09G128200*, *Glyma.09G128300*, and *Glyma.09G128400*) within this block is biosynthetic enzymes, mainly glycosyltransferase involved in isoflavonoid pathway, as well as regulatory genes such as *WRKY* and *MYB* transcription factors (**Table 1**; **Supplementary Tables 3**-**5**). Their likely role as regulatory genes suggests their potential involvement at the transcriptional level in glyceollin elicitation in response to SCN stress (Colinas and Goossens, 2018).

We also found putative genes encoding enzymes involved in the specialized metabolic pathways within the LD blocks of the significant SNPs on chromosomes 3, 13, 15, and 20. The enriched GO category includes the phenylpropanoid metabolic process (GO:0055085,GO:0016021,GO:0008308,GO:0006873), linamarin biosynthesis (GO:0055114,GO:0020037,GO:0016705,GO:0005506), and terpenoid biosynthesis (GO:0016829,GO:0010333,GO:0008152,GO:0000287) (**Supplementary Table 3**). Apart from the biosynthetic enzymes on these chromosomes, we also found transcription factor genes, such as *WRKY*, *MYB*, and *NAC* on chromosomes 3, 9, 13, and 15. For instance, candidate genes within the *WRKY* family transcription factor group include *Glyma.03G176600*, *Glyma.09G129100*, *Glyma.09G127100*, *Glyma.15G139000*, and *Glyma.15G135600*. In the *MYB* transcription factor category, promising candidate genes include *Glyma.09G113000*, *Glyma.09G113100*, and *Glyma.15G134100*. Additionally, the *NAC* transcription factors include *Glyma.13G274300*, *Glyma.13G279900*, and *Glyma.13G280000* as potential candidate genes (**Table 1**, **Supplementary Table 3**).

### 3.3 Metabolic gene clusters identification

We were particularly interested in the candidate genes in the branch from daidzein to glyceollin in the isoflavonoid biosynthesis pathway (Lozovaya et al., 2007). We found that the identified candidate genes on chromosome 9 are clustered together based on our analysis using the PMN plant metabolic cluster viewer, and they fall into two clusters. These two clusters belong to the tailoring enzyme glycosyltransferase within the phenylpropanoid specialized metabolic domain (**Supplementary Table 4**) (Hawkins et al., 2021). Six genes that belong to these two clusters are within the branch of the isoflavonoid biosynthesis pathway. Two of these six genes, *Glyma.09G127200* and *Glyma.09G127300*, are called cluster 1, while the other four (*Glyma.09G127700*, *Glyma.09G128200*, *Glyma.09G128300*, and *Glyma.09G128400*) are called cluster 2 (**Supplementary Table 4**).

Through further investigation of annotation of these candidate genes within the gene clusters (**Supplementary Table 5**), we found the candidate gene *Glyma.09G127200* encodes a glucosyltransferase. Interestingly, the four genes within cluster 2 have a similar functional annotation as *Glyma.09G127200 and Glyma.09G127300* in cluster 1, and all these four genes could be isogenes suggesting their origin from genome duplication events (**Supplementary Table 5**) (Bharadwaj et al., 2021).

### 3.4 Epistatic interactions among all significant SNPs

The results of the epistasis tests for each of the 28 pairwise combinations of the eight significant SNPs are shown in **Table 2**. Three probabilities, all associated with the SNP on chromosome 20, were not estimable (**Table 2**). Among the remaining 25 SNP pairs, 20 show statistical significance. Particularly noticeable is the high significance for all interactions of the SNPs on chromosomes 3, 13, and 15. Three of the six pairs among the four SNPs on chromosome 9, all involving ss715603462, are also statistically significant. In general, therefore, this is evidence for substantial epistasis among these SNPs affecting GVSD.

**Table 2:** Epistasis for the eight significant SNPs.

	Ch9a	Ch9b	Ch9c	Ch9d	Ch13	Ch15	Ch20
**Ch3**	<0.001*	<0.001*	<0.001*	<0.001*	<0.001*	<0.001*	0.002*
**Ch9a**		0.10	0.053	0.007*	<0.001*	<0.001*	0.907
**Ch9b**			0.012*	0.006*	<0.001*	<0.001*	0.835
**Ch9c**				<0.000*	<0.001*	<0.001*	n.e.
**Ch9d**					<0.001*	<0.001*	n.e.
**Ch13**						<0.001*	n.e.
**Ch15**							0.001*

Shown are the probabilities for each pairwise interaction of SNPs. * = P < 0.05 from sequential Bonferroni tests. n.e. = not estimable. Ch3 = ss715585948, Ch9a = ss715603454, Ch9b = ss715603455, Ch9c = ss715603462, Ch9d = ss715603471, Ch13 = ss715615975, Ch15 = ss715620269, Ch20 = ss715636844.

**Figure 3:**
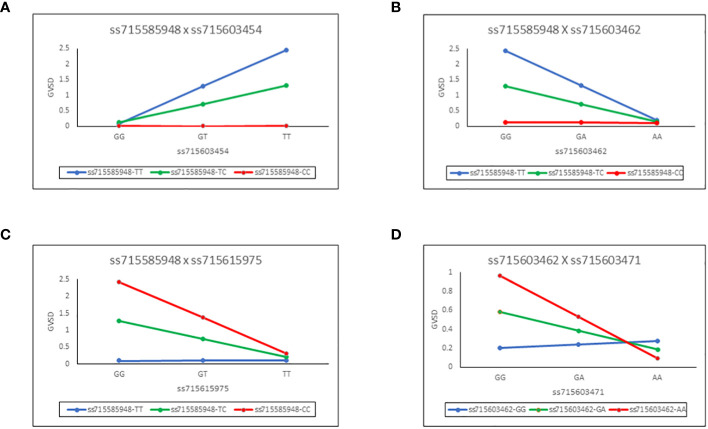
Epistatic interactions of the SNP pairs for each of four chosen combinations. Regression slopes of GVSD on ss715603454 are close to 0 for ss715603454 CC genotypes but are positive for TC and especially TT genotypes **(A)**. Regression slopes of GVSD on ss715603462 are close to 0 for ss715585948 CC genotypes but are negative for TC and especially TT genotypes **(B)**. Regression slopes of GVSD on ss715615975 are close to 0 for ss715585948 TT genotypes but are negative for TC and especially CC genotypes **(C)**. Regression slopes of GVSD on ss715603471 are negative in sign for ss715603462 AA and GA genotypes, but positive in sign for GG genotypes **(D)**.

These epistatic interactions of the SNP pairs are illustrated in **Figure 3** for each of the four chosen combinations. For example, in panel A (**Figure 3A**), it can be seen that regression slopes of GVSD on ss715603454 are close to 0 for ss71585948 CC genotype but are positive for TC and especially TT genotypes. In panel D (**Figure 3D**), regression slopes of GVSD on ss715603471 are negative for ss715603462 AA and GA genotypes but positive for GG genotypes. With no epistasis, these slopes would be expected to be roughly parallel, but in fact, they diverge considerably from parallelism in these four examples, indicating epistasis.

### 3.5 Significant SNPs exhibited extended haplotype homozygosity

**Figure 4:**
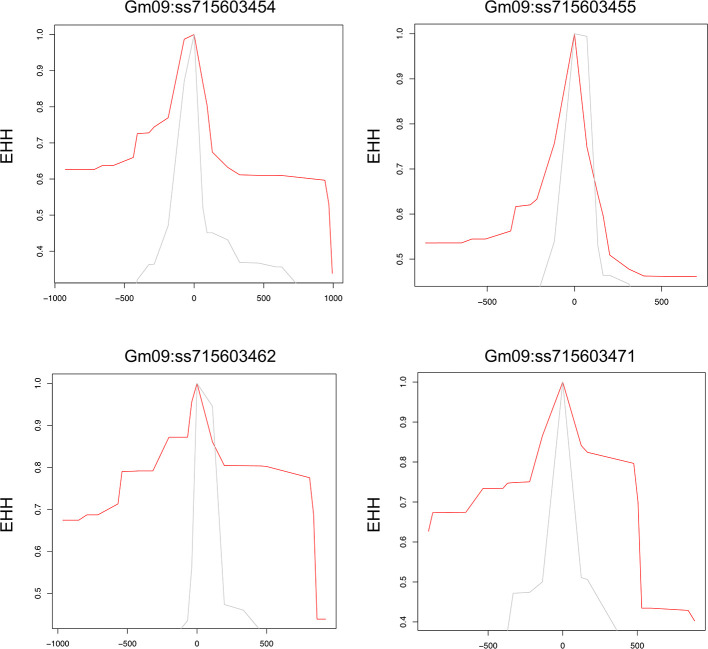
Allele-specific Extended Haplotype Homozygosity (EHH) for the four significant SNPs on chromosome 9. Haplotype lengths are shown flanking the T (red) and G (grey) allele.

To examine allele-specific selection patterns associated with the identified significant SNPs, we conducted an analysis of extended haplotype homozygosity (EHH) for each of these SNPs, as proposed by Sabeti et al. (2002) (Sabeti et al., 2002). The extended homozygosity analysis (EHH) analyses revealed allele specific EHH values of the significant SNPs (ss715603454, ss715603455, ss715603462, and ss715603471) on chromosomes 9 (**Figure 4**). For example, the T allele of ss715603454 showed a much higher EHH value than the G allele. Alleles of significant SNPs on the other chromosomes showed compatible EHH values (**Figure 4**).

## 4 Discussion

### 4.1 Metabolic gene clusters in glyceollin elicitation

Gene clusters have been reported to play important roles in phytochemical diversity in *Arabidopsis*, sorghum, soybean, tomato and rice (Chae et al., 2014; Fan et al., 2020), as well as their roles in important ecological functions in plants i.e., antibacterial, anti-herbivore, antifungal, and insecticidal activities (Polturak and Osbourn, 2021; Polturak et al., 2022). However, their roles in regulating metabolic variation in wild species are relatively less investigated. Even though the isoflavonoid biosynthesis pathway is relatively well studied, the genetic basis of glyceollin elicitation is unclear. Particularly, the contribution, prevalence, and occurrence of gene clusters in plant metabolic diversity are largely unclear. Our mGWAS results suggest there are two probable gene clusters with functionally related but non-homologous genes, which may involve glyceollin elicitation in wild soybean. Thus far, to the best of our knowledge, the genes within these plausible clusters are the first reported candidate genes located on chromosome 9 involved in glyceollin accumulation induced by biotic stimuli in wild soybean. To date, the reported glyceollin biosynthesis genes are located on chromosomes 1, 2, 3, 4, 6, 7, 10, 11, 13, 15, 19 and 20 (Akashi et al., 2009; Yoneyama et al., 2016; Sukumaran et al., 2018; Jahan et al., 2020). Our predicted gene clusters suggest that glyceollin may be synthesized where the enzyme-encoding genes are adjacent to each other on the same chromosome (Chavali and Rhee, 2018). Physical clustering of genes with similar functions can facilitate co-inheritance of alleles with favorable combinations and their coordinated regulations at chromatin level (Osbourn, 2010a; Chu et al., 2011). Besides, such clusters incline to locate in the sub-telomeric regions (Gierl and Frey, 2001; Qi et al., 2004; Sakamoto et al., 2004), near the ends of chromosomes that are known to harbor mutations. For example, an examination of the complete genome sequence revealed that the maize *DIMBOA* cluster is located close to the end of chromosome 4 (Farman, 2007; Jonczyk et al., 2008). Thus, identifying the positions of the genes can contribute to inferences of possible mechanisms underlying chemical diversity in natural populations.

Beyond gene clusters playing critical roles in phytochemical diversity, tailoring enzymes, such as methyltransferases, glycosyltransferases, *CYPs*, dehydrogenases/reductases, and acyltransferases, are reported to be responsible for modifying the chemical backbone of specialized metabolites (Osbourn, 2010b). The genes in these two plausible clusters are annotated with tailoring or regulating glycosyltransferase enzymes. One of the common plant defense mechanisms involves glycosylation of secondary metabolites with these enzymes (Mylona et al., 2008). Therefore, the clustering of the genes encoding glycosyltransferase on chromosome 9 might be very critical in the formation of glyceollin, the stress-induced (i.e., SCN stress in our study) protective compounds in legumes. For example, the cyclic hydroxamic acid *(DIBOA)* in maize (Frey et al., 1997; Gierl and Frey, 2001), the triterpene avenacin in oat (Qi et al., 2004; Qi et al., 2006; Field and Osbourn, 2008; Mugford et al., 2009), and two gene clusters associated with diterpene (momilactone and phytocassane) synthesis in rice, which may be pre-formed or synthesized after stress elicitation for plant defense. Disruption of such gene clusters may compromise pest and disease resistance and lead to the accumulation of toxic pathway intermediates (Chu et al., 2011). In the multi-step plant specialized metabolic pathways, rapid adaptation to a particular environmental niche could result in highly diverse and rapidly evolving metabolic gene clusters (Osbourn and Field, 2009). Hence, the level of conservation of the identified gene clusters across different legume species may shed light on the evolutionary insight of these clusters (Field and Osbourn, 2008). Synthetic biology and functional genetics can further help investigate the organization and contribution of these clusters in metabolite diversity, as well as decipher the mechanism of adaptive evolution and genome plasticity (Osbourn, 2010b; Chu et al., 2011).

### 4.2 Plausible transcriptional factors in glyceollin elicitation

Advancement of genetics, genomics, and bioinformatic approaches facilitate the prediction and identification of a large number of genes, including transcription factors associated with plant-specialized metabolic pathways (Anarat-Cappillino and Sattely, 2014; Moore et al., 2019). However, the transcriptional regulators of specialized metabolism are less well characterized (Shoji and Yuan, 2021). The regulation of plant-specialized metabolic pathways is dynamic, reflecting the inherent adaptability of these pathways to the ever-changing environment. Such regulation generally occurs at transcription level, and thus, it requires coordinated regulation mediated by transcription factors (TFs) (Colinas and Goossens, 2018; Shoji, 2019). For instance, *MYB* and basic helix-loop-helix (bHLH) TF family genes were reported to regulate anthocyanin and related flavonoid biosynthetic pathways in a wide range of species (Chezem and Clay, 2016). Moreover, significant modifications of these regulatory genes give rise to the vast diversity in plant specialized metabolism (Huang et al., 2018; Springer et al., 2019).

It is possible that transcription factors, such as *MYB* and *WRKY* TFs on chromosome 9, may influence glyceollin elicitation. The regulation of glyceollin elicitation with SCN stress may involve a highly complex interplay among multiple genes and pathways. Previous studies reported that gene families of transcription factors, such as *NAC*, *MYB*, *bHLH*, and *WRKY*, exhibited conservative patterns among *Arabidopsis*, cotton, grapevine, maize, and rice (Xu et al., 2004; Zheng et al., 2006; Saga et al., 2012; Ibraheem et al., 2015; Yamamura et al., 2015; Ogawa et al., 2017). These plant species produce various phytoalexins, such as indole alkaloids, terpenoid aldehydes, stilbenoids, deoxyanthocyanidins, and momilactones/phytocassanes, respectively. The investigation of TFs binding promoter regions can give insights if the pathways are co-opted into stress-inducible regulation by the respective TFs such as *NAC* TF gene *GmNAC42–1* and *MYB* TF gene *GmMYB29A2* regulates glyceollin biosynthesis (Jahan et al., 2019; Jahan et al., 2020). The transcription factor gene *GmNAC42-1* plays a crucial role as a positive regulator in glyceollin biosynthesis. Jahan and colleagues (Jahan et al., 2019) showed that elevating the expression of *GmNAC42-1* in hairy roots has the potential to amplify glyceollin yields by more than tenfold when elicited. Furthermore, the TF gene *GmMYB29A2*, as identified by Jahan et al. (2020) (Jahan et al., 2020), plays a crucial role in both the accumulation of glyceollin I and the expression of resistance against *Phytophthora*. It would be intriguing to explore whether the transcription factor genes we’ve identified exhibit homology across different plant species. The homology of TFs among different plant species can facilitate metabolic engineering of a wide variety of crop plants to produce phytoalexins in greater amounts (Ahmed and Kovinich, 2021).

In addition to enzyme-encoding genes, TF genes can also be found as gene clusters. For example, the gene cluster of TF *ERF* (jasmonate (JA)- responsive ethylene response factor) consists of five *ERF* genes in tomato (Cárdenas et al., 2016; Thagun et al., 2016), eight in potato (Cárdenas et al., 2016), five in tobacco (Kajikawa et al., 2017), five in *C. roseus* (Singh et al., 2020), four in *Calotropis gigantea* (Singh et al., 2020), and four in *Glesemium sempervirens* (Singh et al., 2020). Besides, TFs involved in plant specialized metabolism can be found in arrays (Zhou et al., 2016; Shoji and Yuan, 2021). Thus, it is possible that the TFs we identified are located in the same genomic neighborhood as arrays or biosynthetic gene clusters (BGCs). The co-regulation hypothesis of gene clusters poses that clustering of TFs can co-regulate genes in a pathway. Although co-regulation of metabolic pathways also occurs un-clustered, clustering may accelerate the recruitment of genes into a regulon (Wisecaver et al., 2017; Smit and Lichman, 2022).

### 4.3 Epistasis and plausible selection on glyceollin elicitation

Metabolic traits have been reported with low heritability due to environmental effects on their accumulations (Rowe et al., 2008). Recent studies have shown strong epistatic interactions of genes influencing variation of plant specialized metabolites, which may impact fitness in the field (Brachi et al., 2015; Kerwin et al., 2015; Kerwin et al., 2017). For example, numerous epistatic interactions influence the highly complex genetic architecture responsible for *Arabidopsis* metabolism (Kliebenstein, 2001; Kliebenstein et al., 2001). Moreover, a mixture of positive and negative epistatic interactions can assist identifying significant QTLs located within a biosynthetic pathway (Rowe et al., 2008). Compared to expression regulations, the power of epistasis in metabolomics is that they can better indicate the interconnectedness of metabolites within the metabolic pathway (Fell and Wagner, 2000; Jeong et al., 2000; Arita, 2004). The widespread interactive effects found among our identified significant SNPs affecting targeted metabolic traits may be a consequence of the interconvertibility between daidzein and glyceollin. As an example, the study conducted by Farrell et al. (2017) demonstrated that there is an augmentation in the biosynthesis of glyceollin I from daidzein when there is an elevation in the degradation of 6”-O-malonyldaidzin, an isoflavone conjugate produced from daidzein (Farrell et al., 2017).

Genes containing causal variation for plant defensive compounds may influence field fitness and thus are likely under natural selection (Kroymann, 2011). For example, Benderoth et al. (2006) detected positive selection in glucosinolate diversification in *Arabidopsis thaliana* and its relatives (Benderoth et al., 2006). Prasad et al. (2012) showed positive selection for a mutation on a metabolic pathway gene could enhance resistance to herbivory in natural populations of a rocky mountain cress species (Prasad et al., 2012). We detected strong signals of selection on the SNPs significantly associated with glyceollin phenotypes with EHH and LD analyses (**Figure 4**; **Figure 2B**). For example, the LD surrounding the significant SNP ss715603454 that is next to the identified gene clusters is more extensive, suggesting strong selection in this region (**Figure 2B**). Meanwhile, the two alleles of this significant SNP, G and T, showed different EHH values, with T exhibiting much longer haplotype homozygosity. This indicates that this T allele may be under recent positive selection. Interestingly, the T allele is significantly associated with higher elicitation of glyceollin and has a higher frequency in South Korea (**Figures 2C, D**). The allele specific EHH pattern and their geographic distribution may be due to heterogeneous selection pressure in nature.

### 4.4 Perspectives and future directions of our study

Plant specialized metabolites exhibit extreme quantitative and qualitative variation. Therefore, high-throughput metabolite profiling, such as LC-MS analysis coupled with GWAS (as applied here) can facilitate understanding the genetic contributions to metabolic diversity in natural populations. A common assumption is that biological variables or traits should show a normal distribution, and skewed data may indicate measurement error. However, the scenario is different in metabolomics, especially in secondary metabolism. For instance, a ratio of two related compounds, rather than their separate values, may provide a comprehensive understanding of the underlying enzymatic process (Byrne et al., 1996; McMullen et al., 1998; Yencho et al., 1998; Kliebenstein, 2001; Kliebenstein et al., 2001; Kliebenstein, 2007; Chan et al., 2011; Petersen et al., 2012; Prasad et al., 2012). We used a ratio of glyceollin and daidzein concentrations as the phenotypic trait for our association study. The use of a metabolic ratio also may produce: (1) a reduction in the variability of the data collected for the biological replicates and thus increase statistical power, and (2) a reduction in overall noise in the dataset by canceling out systemic experimental errors. Most importantly for our purposes, the glyceollin to daidzein metabolite ratio is correlated to the corresponding reaction rate under optimal steady-state assumptions, as this metabolite pair is connected in the phenylpropanoid biosynthetic pathway (Suhre et al., 2011; Petersen et al., 2012).

The natural world has a lot to offer in tackling diseases and global food scarcity. There is a need to develop new medicines and future value-increased food by unlocking the uncharted gene pools of wild plants. Our chosen study system crop wild relative of soybean poses much higher and underexplored genetic diversity than its domesticated descendants. Given that glyceollin is produced in trace amounts, it is an exciting challenge to define the plant metabolic gene clusters and transcriptional regulators in the glyceollin biosynthesis pathway. Besides complex cancer treatment and therapies, the rise of different types of tumors and tumor subtypes urges the need for new drugs. Along with glyceollin’s role in plant defense, it has been well-documented for anti-cancer activities. Our follow-up studies will apply transcriptomics and functional validation of the candidate genes, which can expand our focus to explore associations of genes in clusters to understand their involvement in regulating glyceollin biosynthesis at the systems level. As phytochemical variation can be caused by both structural genes and their expression differences, it will be interesting to explore the role of pathway-specific regulators (i.e., transcription factors) in glyceollin elicitation (Osbourn, 2010b). Our results suggest that improving our fundamental knowledge of plant specialized metabolic gene clusters and regulators will facilitate metabolic engineering with improved metabolic traits for sustainable agriculture and novel pharmaceuticals.

## Data availability statement

The datasets presented in this study can be found in online repositories. The names of the repository/repositories and accession number(s) can be found in the article/**Supplementary Material**.

## Author contributions

B-HS conceived the idea and initiated the project. FY, HZ, LL, BW, JW, RR, and CB performed experiments and analyzed data. FY, HZ, LL, BW, and B-HS wrote and improved the manuscript. FY and HZ contributed equally. All authors contributed to the article and approved the submitted version.
