# Supplementary material for: Genetic basis and selection of glyceollin elicitation in wild soybean
Source: Front Plant Sci. 2024 Feb 28;15:1240981. doi: 10.3389/fpls.2024.1240981 (PMC10933117; doi:10.3389/fpls.2024.1240981)
Supplement: Supplementary file 1 [file DataSheet_1.pdf]

Supplementary Material

Genetic basis and selection of glyceollin elicitation in wild soybean

Farida Yasmin[1†], Hengyou Zhang[1,2†*], Larry Leamy[1], Baosheng Wang[3,4], Jason Winnike[5], Robert W. Reid[6], Cory R. Brouwer[6] and Bao-Hua Song[1*]

*** Correspondence:** Bao-Hua Song: bsong5@uncc.edu; Hengyou Zhang: zhanghengyou@iga.ac.cn

1 Supplementary Figures

Figure S1:

Figure S2:

2 Supplementary Figures and Tables

Table S1

Table S2

Table S3

Table S4

Table S5

1. Supplementary Figures

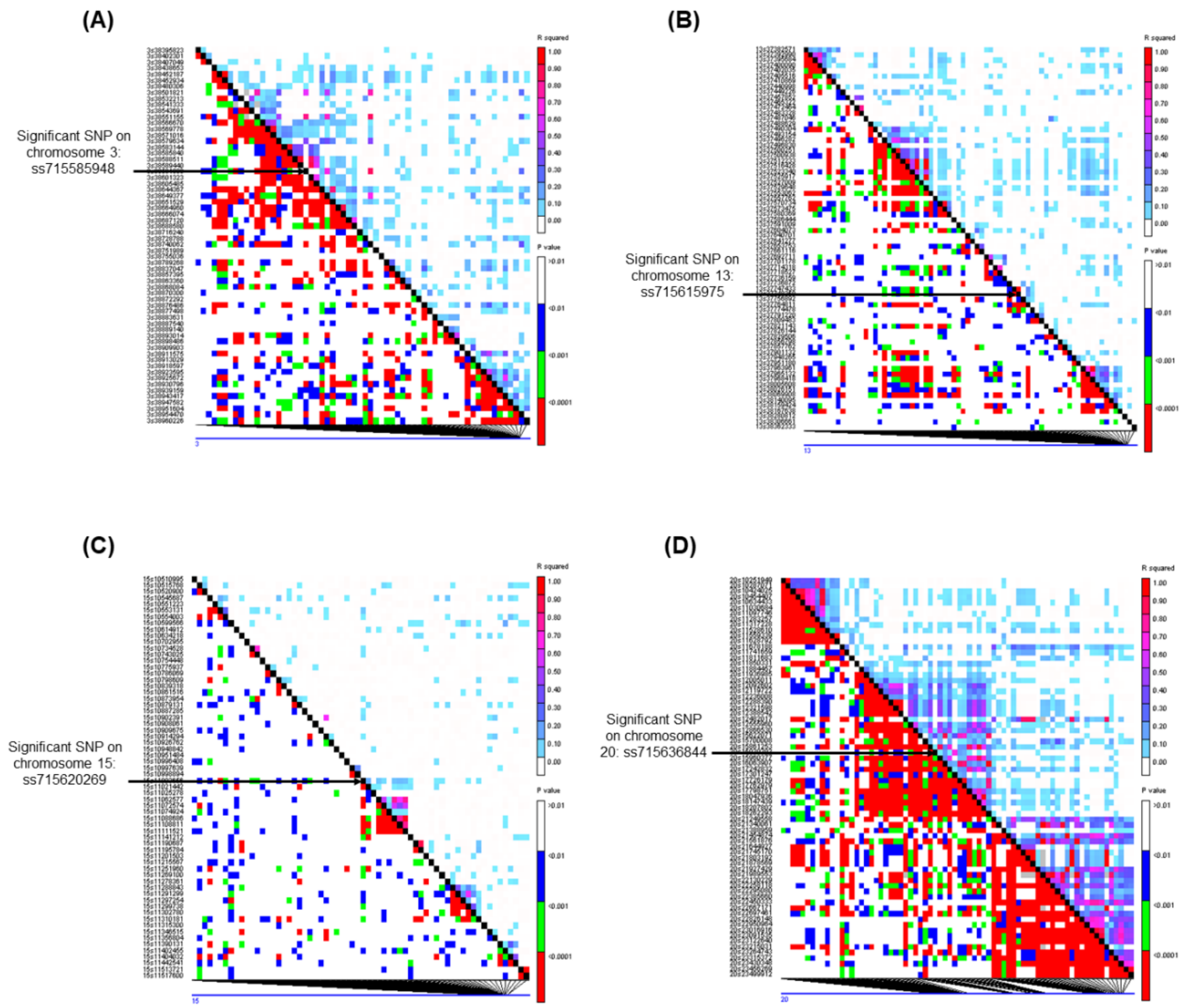

Figure S1. Significant SNPs ss715585948 **(A)**, ss715615975 **(B)**, ss715620269 **(C)**, and ss715636844 **(D)** on chromosomes 3, 13, 15, and 20, respectively, show narrow LD blocks.

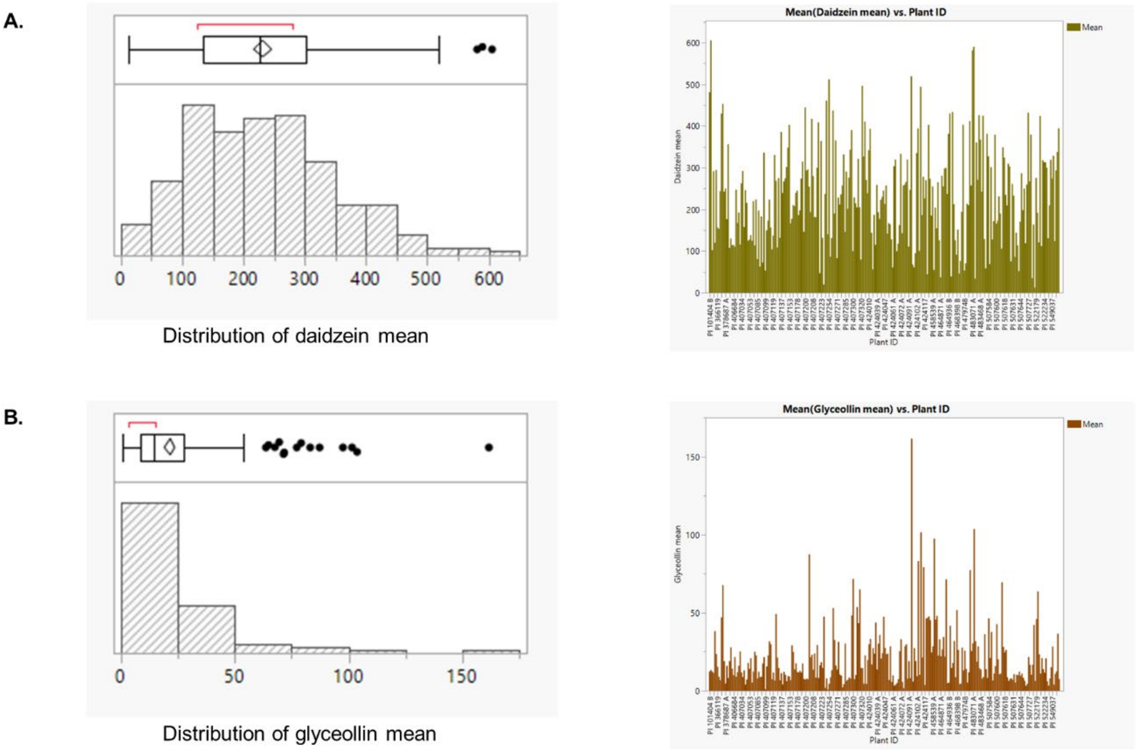

Figure S2. Representation of metabolite data generated and analyzed in this study. Distribution of daidzein means and visual representation of daidzein mean for each *G. soja* accession utilized in this study **(A)**. Distribution of glyceollin means and visual representation of glyceollin mean for each *G. soja* accession utilized in this study **(B)**.

2 Supplementary Tables

Table S1: Wild soybean ecotypes used and metabolite data collected in this study.

Genotype ID	Species Name	Origin*	Latitude	Longitude	Daidzein (DZN)	Glyceollin (GLY)	GVSD
					Mean	Mean	GLY/DZN
PI101404B	Glyine soja	CN	46.20	126.07	481.66	12.28	0.025
PI339732	Glyine soja	SK	37.31	128.54	120.07	38.23	0.318
PI366120	Glyine soja	JP	39.53	140.38	153.55	9.04	0.059
PI366122	Glyine soja				429.95	46.99	0.109
PI366123	Glyine soja	JP	39.70	141.25	452.9	67.63	0.149
PI406684	Glyine soja	JP	42.87	142.44	114.75	9.98	0.087
PI407037	Glyine soja	JP	39.70	140.73	291.78	8.76	0.03
PI407047	Glyine soja	JP	39.72	140.07	246.75	3.88	0.016
PI407050	Glyine soja	JP	39.72	141.14	124.78	17.82	0.143
PI407053	Glyine soja	JP	36.10	137.97	127.95	14.23	0.111
PI407089	Glyine soja	JP	34.80	134.98	197	12.23	0.062
PI407097	Glyine soja	JP	34.76	135.16	336.6	21.49	0.064
PI407120	Glyine soja	JP	35.00	135.00	330.83	6.6	0.02
PI407167	Glyine soja	SK	37.28	127.12	210.86	24.92	0.118
PI407174	Glyine soja	SK	37.20	127.44	239.72	17.63	0.074
PI407198	Glyine soja	SK	37.62	127.82	146.65	7.72	0.053
PI407201	Glyine soja	SK	37.44	127.98	293.56	7.8	0.027
PI407202	Glyine soja	SK	37.50	127.98	296.04	7.49	0.025
PI407217	Glyine soja	SK	36.96	127.85	299.71	29.32	0.098
PI407221	Glyine soja	SK	36.88	127.65	408.81	8.2	0.02
PI407246	Glyine soja	SK	35.68	128.75	237.14	1.9	0.008
PI407249	Glyine soja	SK	35.67	128.66	460.95	8.03	0.017
PI407254	Glyine soja	SK	35.52	128.78	512.02	4.62	0.009
PI407267	Glyine soja	SK	35.40	128.63	365.62	16.17	0.044
PI407271	Glyine soja	SK	35.56	126.87	82.87	13.69	0.165
PI407275	Glyine soja	SK	37.43	126.99	236.38	9.75	0.041
PI407278	Glyine soja	SK	37.57	127.23	257.34	2.31	0.009
PI407302	Glyine soja	CN	32.06	118.85	228.88	7.49	0.033
PI407304	Glyine soja	CN	31.02	121.41	214.35	10.08	0.047
PI424059B	Glyine soja	SK	38.25	127.39	128.16	5.71	0.045
PI424063	Glyine soja	SK	38.16	127.41	303.37	3.33	0.011
PI424064	Glyine soja	SK	38.08	128.05	319.62	3.97	0.012
PI424088	Glyine soja	SK	37.13	128.22	319.47	7.86	0.025
PI424093	Glyine soja	SK	36.99	128.36	246.99	8.13	0.033

PI424102A	Glyine soja	SK	36.50	128.15	335.17	9.45	0.028
PI424117	Glyine soja	SK	35.92	129.00	226.5	3.65	0.016
PI468396B	Glyine soja	CN	36.42	113.20	433.76	17.8	0.041
PI468397A	Glyine soja	CN	37.73	112.47	212.67	31.95	0.15
PI468398B	Glyine soja	CN	37.76	112.42	91.17	51.69	0.567
PI468399B	Glyine soja	CN	37.04	118.91	152.02	26.08	0.172
PI483466	Glyine soja	CN	36.24	116.84	478.83	19	0.04
PI483468A	Glyine soja	CN	33.51	115.23	367.35	13.65	0.037
PI487430	Glyine soja	JP	42.60	142.10	242.74	7.84	0.032
PI507582	Glyine soja	JP	40.68	141.36	381.5	8.28	0.022
PI507632	Glyine soja	JP	34.50	135.80	232.88	10.64	0.046
PI507644	Glyine soja	JP	33.84	132.75	170.84	9.62	0.056
PI508067	Glyine soja	JP	42.37	142.41	163.91	42.16	0.257
PI522179	Glyine soja	CN			275.57	46.06	0.167
PI522180	Glyine soja	CN	50.21	126.82	191.98	63.71	0.332
PI549037	Glyine soja	CN	40.55	124.07	274.15	28.39	0.104
PI549046	Glyine soja	CN	40.71	125.04	123.74	10.92	0.088
PI562544	Glyine soja	SK	36.85	126.93	293.38	12.38	0.042
PI562550	Glyine soja	SK	36.57	126.68	338.03	36.65	0.108
PI578345	Glyine soja				394.63	7.59	0.019
PI163453	Glyine soja	CN			102.5	12.28	0.12
PI366121	Glyine soja				243.61	6.74	0.028
PI378695A	Glyine soja	JP			356.08	8.27	0.023
PI378701A	Glyine soja	JP	39.70	141.20	129.97	27.83	0.214
PI407030	Glyine soja	JP	39.55	140.36	192.77	16.09	0.083
PI407034	Glyine soja	JP	39.50	140.36	262.49	11.82	0.045
PI407124	Glyine soja	JP	35.00	135.00	108.38	21.3	0.197
PI407200	Glyine soja	SK	37.44	127.98	444.83	7.14	0.016
PI407279	Glyine soja				331.73	4.28	0.013
PI407296	Glyine soja	CN	41.64	123.48	343.46	7.74	0.023
PI423993	Glyine soja	RU	52.98	127.36	410.68	22.75	0.055
PI423996	Glyine soja	RU	52.98	127.36	270.76	4.46	0.016
PI464934	Glyine soja	CN			237	4.97	0.021
PI464936B	Glyine soja	CN			381.53	5.23	0.014
PI464937A	Glyine soja	CN			429.83	41.73	0.097
PI423988	Glyine soja	RU	52.98	127.36	326.95	4.55	0.014
PI479749	Glyine soja	CN			70.48	14.11	0.2
PI479751	Glyine soja	CN			210.36	11.88	0.056
PI483466	Glyine soja	CN	36.24	116.84	373.82	10.16	0.027

PI504287A	Glyine soja	JP	39.72	141.14	424.78	8.76	0.021
PI507727	Glyine soja	RU			432.03	21.61	0.05
PI507787	Glyine soja	RU			267.03	16.63	0.062
PI507798	Glyine soja	RU			379.29	10.27	0.027
PI508066	Glyine soja	JP	42.37	142.41	34.89	16.71	0.479
PI508069	Glyine soja	JP	42.37	142.41	12.69	6.39	0.503
PI522211B	Glyine soja	RU	45.00	135.00	318.49	13.84	0.043
PI522234	Glyine soja	RU	45.00	135.00	313.78	11.66	0.037
PI135624	Glyine soja	CN	45.83	126.69	605.46	13.36	0.022
PI339731	Glyine soja	SK	37.91	128.04	291.69	11.41	0.039
PI339735A	Glyine soja	SK	37.44	128.40	294.74	23.58	0.08
PI366119	Glyine soja	JP	34.85	136.92	157.15	15.37	0.098
PI378685	Glyine soja	JP	34.03	132.85	242.67	19.04	0.078
PI378687A	Glyine soja	JP	32.89	130.74	250.1	4.69	0.019
PI378689	Glyine soja	JP	37.10	138.25	177	16.04	0.091
PI378700	Glyine soja	JP	38.48	140.38	107.78	19.05	0.177
PI378702	Glyine soja	JP	39.70	141.20	114.53	14.23	0.124
PI407020	Glyine soja	JP	39.55	140.36	110.76	21.58	0.195
PI407026	Glyine soja	JP	39.55	140.36	247.46	8.97	0.036
PI407029	Glyine soja	JP	39.72	140.07	167.62	11.83	0.071
PI407032B	Glyine soja	JP	39.55	140.36	116.14	25.13	0.216
PI407044	Glyine soja	JP	39.57	140.42	158.08	13.11	0.083
PI407048	Glyine soja	JP	39.72	141.14	215.76	7.27	0.034
PI407055	Glyine soja	JP	35.00	138.00	138.77	20.97	0.151
PI407060	Glyine soja	JP	34.82	136.92	124.65	5.27	0.042
PI407063	Glyine soja	JP	34.85	136.87	219.83	12.3	0.056
PI407069	Glyine soja	JP	34.77	136.90	113.59	25.01	0.22
PI407072	Glyine soja	JP	34.96	137.61	224.09	22.73	0.101
PI407085	Glyine soja				81.18	7.94	0.098
PI407092	Glyine soja	JP	35.13	134.96	62.73	8.77	0.14
PI407094	Glyine soja	JP	34.88	135.19	182.95	9.01	0.049
PI407096	Glyine soja	JP	34.92	135.23	72.82	17.51	0.24
PI407099	Glyine soja				53.1	1.01	0.019
PI407100	Glyine soja	JP	34.76	135.16	149.74	15.6	0.104
PI407102	Glyine soja	JP	34.76	135.16	172.99	22.66	0.131
PI407107	Glyine soja	JP	34.76	135.16	223.44	31.6	0.141
PI407109	Glyine soja	JP	34.76	135.16	156.86	29.86	0.19
PI407113	Glyine soja				104.36	7.42	0.071
PI407119	Glyine soja	JP	35.00	135.00	137.38	11.78	0.086

PI407121	Glyine soja	JP	35.00	135.00	268.47	49.25	0.183
PI407126	Glyine soja	JP	34.65	133.92	274.94	14.69	0.053
PI407131	Glyine soja	JP	32.88	130.97	132.24	6.7	0.051
PI407137	Glyine soja				386.04	11.31	0.029
PI407142	Glyine soja	JP	32.89	130.59	239.53	4.01	0.017
PI407145	Glyine soja	JP	32.89	130.59	267.22	12.14	0.045
PI407147	Glyine soja	JP	32.92	130.59	274.61	10.24	0.037
PI407149	Glyine soja	JP	32.83	130.72	301.83	6.29	0.021
PI407151	Glyine soja	JP	32.83	130.72	348.18	9.44	0.027
PI407153	Glyine soja	JP	32.83	130.72	402.59	9.21	0.023
PI407155	Glyine soja	JP	32.88	130.74	166.19	6.77	0.041
PI407159	Glyine soja	SK	37.28	127.11	177.23	29.13	0.164
PI407172	Glyine soja	SK	37.23	127.28	207.74	13.78	0.066
PI407178	Glyine soja	SK	37.35	127.44	244.94	12.1	0.049
PI407187	Glyine soja	SK	37.12	127.06	186.62	11.77	0.063
PI407191	Glyine soja	SK	37.28	127.02	197.66	12.84	0.065
PI407193	Glyine soja	SK	37.75	127.80	273.85	11.79	0.043
PI407194	Glyine soja	SK	37.82	127.75	314.59	17.52	0.056
PI407203	Glyine soja	SK	37.29	127.92	254.93	87.28	0.342
PI407205	Glyine soja	SK	37.28	127.91	193.68	21.45	0.111
PI407207	Glyine soja	SK	37.16	127.89	417.13	23.04	0.055
PI407208	Glyine soja	SK	37.16	127.89	279.89	13.56	0.048
PI407211	Glyine soja	SK	37.08	127.89	182.04	23.93	0.131
PI407214	Glyine soja	SK	37.05	127.95	181.46	8.44	0.047
PI407222	Glyine soja	SK	36.86	127.63	47.21	16.24	0.344
PI407223	Glyine soja	SK	36.76	127.55	363.84	18.27	0.05
PI407233	Glyine soja				131.46	14.67	0.112
PI407236	Glyine soja	SK	36.65	127.27	19.81	47.51	2.398
PI407252	Glyine soja	SK	35.51	128.77	140.21	0.82	0.006
PI407256	Glyine soja	SK	35.56	128.83	86.91	8.71	0.1
PI407258	Glyine soja	SK	35.55	128.75	131.4	13.25	0.101
PI407260	Glyine soja	SK	35.61	128.54	437.48	52.94	0.121
PI407261	Glyine soja	SK	35.58	128.51	190.26	32.54	0.171
PI407272	Glyine soja	SK	35.52	126.90	228.67	31.35	0.137
PI407274	Glyine soja	SK	35.51	126.91	211.85	10.49	0.05
PI407285	Glyine soja	JP	35.59	139.35	146.2	30.36	0.208
PI407290	Glyine soja	CN	43.51	124.81	290.63	6.43	0.022
PI407292	Glyine soja	CN	43.85	125.31	201.08	7.1	0.035
PI407294	Glyine soja	CN	43.85	125.31	276.25	8.61	0.031

PI407298	Glyine soja	CN	41.64	123.48	390.28	48.21	0.124
PI407300	Glyine soja	CN	32.06	118.85	100.11	71.74	0.717
PI407310	Glyine soja	SK	37.08	127.42	204.82	53.66	0.262
PI407312	Glyine soja	SK	36.87	127.54	321.37	43.39	0.135
PI407319	Glyine soja	SK	36.47	127.70	205.31	64.89	0.316
PI407320	Glyine soja	SK	37.00	127.58	79.55	14.64	0.184
PI407322	Glyine soja	SK	36.33	127.53	496.39	14.5	0.029
PI424006A	Glyine soja				238.68	19.66	0.082
PI424010	Glyine soja	SK	37.23	126.97	342.33	29.89	0.087
PI424012	Glyine soja	SK	37.23	126.97	393.5	33.31	0.085
PI424017A	Glyine soja	SK	38.09	127.08	144.08	16.88	0.117
PI424022A	Glyine soja	SK	38.02	127.23	56.07	27.34	0.488
PI424023	Glyine soja	SK	38.05	127.26	187.16	23.81	0.127
PI424027B	Glyine soja	SK	38.07	127.30	115.34	43.69	0.379
PI424039A	Glyine soja	SK	37.56	127.54	259.01	13.69	0.053
PI424040	Glyine soja	SK	37.49	127.65	193.3	30.2	0.156
PI424042	Glyine soja	SK	37.54	127.33	177.44	35.72	0.201
PI424043A	Glyine soja	SK	37.55	127.26	223.03	20.69	0.093
PI424044	Glyine soja	SK	37.55	127.26	229.95	24.13	0.105
PI424046A	Glyine soja	SK	37.59	127.22	245.34	47.47	0.194
PI424047	Glyine soja	SK	37.61	127.21	213.04	27.4	0.129
PI424051B	Glyine soja	SK	37.65	127.19	257.25	23.44	0.091
PI424052	Glyine soja	SK	37.64	127.21	141.83	23.79	0.168
PI424056	Glyine soja	SK	38.13	127.35	166.78	6.86	0.041
PI424058	Glyine soja	SK	38.18	127.36	164.37	28.44	0.173
PI424061A	Glyine soja	SK	37.87	127.75	61.11	9.92	0.162
PI424067	Glyine soja	SK	38.12	128.22	100.04	5.01	0.05
PI424068	Glyine soja	SK	38.07	128.17	117.81	7.44	0.063
PI424070A	Glyine soja	SK	37.68	127.88	162.66	25.48	0.157
PI424072A	Glyine soja	SK	39.49	128.00	333.33	33.03	0.099
PI424074	Glyine soja	SK	37.48	128.03	142.88	5.68	0.04
PI424080	Glyine soja	SK	37.50	128.85	257.52	1.71	0.007
PI424085A	Glyine soja	SK	37.64	128.55	259.77	28.51	0.11
PI424086	Glyine soja	SK	37.17	128.27	265.98	29.75	0.112
PI424091A	Glyine soja	SK	37.08	128.26	111.16	19.06	0.171
PI424094	Glyine soja				519.36	161.63	0.311
PI424095	Glyine soja	SK	36.23	127.91	68.3	5.93	0.087
PI424100A	Glyine soja	SK	36.33	128.12	61.49	27.32	0.444
PI424101	Glyine soja	SK	36.34	128.13	94.77	19.07	0.201

PI424104	Glyine soja	SK	36.66	128.12	394.26	83.15	0.211
PI424105	Glyine soja	SK	36.59	128.19	100.14	10.22	0.102
PI424110	Glyine soja	SK	35.77	128.81	494.44	101.53	0.205
PI424112	Glyine soja	SK	36.42	129.08	186.37	21.25	0.114
PI424115A	Glyine soja	SK	35.99	128.91	278.15	79.17	0.285
PI424123	Glyine soja	SK	35.71	129.21	270.12	46.39	0.172
PI424125	Glyine soja	SK	35.49	126.90	44.57	46.79	1.05
PI447004	Glyine soja	CN	42.50	126.83	402.61	47.67	0.118
PI458535	Glyine soja	CN	48.27	126.60	273.47	45.29	0.166
PI458536	Glyine soja	CN	46.86	126.85	185.13	24.56	0.133
PI458539A	Glyine soja	CN	48.82	128.41	255.09	28.57	0.112
PI458540A	Glyine soja	CN	47.02	133.71	54.94	97.53	1.775
PI464867	Glyine soja	CN	50.21	126.82	204.64	45.6	0.223
PI464868A	Glyine soja	CN	48.82	128.41	154.82	47.96	0.31
PI464869A	Glyine soja	CN	46.86	126.85	265.56	22.57	0.085
PI464870	Glyine soja	CN	48.48	127.97	125.82	32.99	0.262
PI464871A	Glyine soja	CN	48.48	127.97	37.42	22.15	0.592
PI464889A	Glyine soja	CN	43.89	124.66	280.3	26.41	0.094
PI464890B	Glyine soja	CN	44.23	126.00	255.16	34.38	0.135
PI464892	Glyine soja	CN			297.98	21.88	0.073
PI464929A	Glyine soja	CN	40.80	122.78	299.85	71.44	0.238
PI464939A	Glyine soja	CN			39.55	14.6	0.369
PI468398A	Glyine soja	CN	37.76	112.42	126.02	8.62	0.068
PI468400A	Glyine soja	CN	37.33	106.04	45.89	4.25	0.093
PI468916	Glyine soja	CN	41.20	122.34	76.58	4.71	0.062
PI479746A	Glyine soja	CN			194.32	27.69	0.143
PI479747	Glyine soja	CN			403.34	13.11	0.033
PI479748	Glyine soja	CN			53.34	25.7	0.482
PI479750	Glyine soja	CN			213.53	5.28	0.025
PI479753A	Glyine soja	CN	43.63	126.50	411.71	77.25	0.188
PI479767	Glyine soja	CN	48.48	127.97	257.23	25.62	0.1
PI483071A	Glyine soja	CN	36.83	120.75	581.21	30.4	0.052
PI483461	Glyine soja	CN	41.54	117.56	589.8	103.73	0.176
PI483463	Glyine soja	CN	38.78	113.42	34.16	31.71	0.928
PI483464A	Glyine soja	CN	38.80	106.67	360.6	18.76	0.052
PI483465	Glyine soja	CN	34.88	110.01	271.14	28.48	0.105
PI504289	Glyine soja	JP	39.33	141.00	129.38	36.33	0.281
PI507581	Glyine soja	JP	40.63	140.60	58.68	13.89	0.237
PI507584	Glyine soja	JP	40.68	141.36	327.56	46.45	0.142

PI507585	Glyine soja	JP	40.68	141.36	68.93	21.01	0.305
PI507590A	Glyine soja	JP	39.67	140.25	301.26	37.62	0.125
PI507590B	Glyine soja	JP	39.67	140.25	127.89	6.57	0.051
PI507591	Glyine soja	JP	39.70	140.73	171.88	11.09	0.065
PI507599	Glyine soja	JP	35.86	140.30	379.13	15.83	0.042
PI507600	Glyine soja	JP	36.33	140.29	165.74	42.7	0.258
PI507601A	Glyine soja	JP	36.33	140.29	172.76	20.13	0.117
PI507605	Glyine soja	JP	36.37	140.48	231.23	10.63	0.046
PI507606	Glyine soja	JP	36.32	139.58	190.19	10.99	0.058
PI507607	Glyine soja	JP	36.32	139.58	106.27	69.53	0.654
PI507617	Glyine soja	JP	40.06	124.56	349.29	28.11	0.08
PI507618	Glyine soja	JP	40.06	124.56	324.33	24.72	0.076
PI507619A	Glyine soja	JP	40.06	124.56	234.91	26.12	0.111
PI507621	Glyine soja	JP	36.60	138.03	209.61	8.68	0.041
PI507626	Glyine soja	JP	34.77	137.38	309.46	6.47	0.021
PI507627	Glyine soja	JP	34.94	137.24	303.05	7.68	0.025
PI507628	Glyine soja	JP	34.77	137.38	75.77	8.06	0.106
PI507631	Glyine soja	JP	34.53	135.95	261.3	7.06	0.027
PI507633	Glyine soja	JP	35.24	135.46	86.49	5.27	0.061
PI507639	Glyine soja	JP	34.85	134.93	143.81	10.2	0.071
PI507640	Glyine soja	JP	34.82	135.42	113.51	9.97	0.088
PI507641	Glyine soja	JP	35.40	134.77	52.04	12.21	0.235
PI507653	Glyine soja	JP	33.87	130.75	286.46	11.11	0.039
PI507655	Glyine soja	JP	33.00	129.50	198.54	8.3	0.042
PI507658	Glyine soja	JP	32.08	130.37	249.79	6.49	0.026
PI507662	Glyine soja	JP	31.33	130.93	118.79	3.28	0.028
PI507665	Glyine soja	JP	32.88	131.10	259.76	4.48	0.017
PI522181	Glyine soja	CN	48.82	128.41	120.9	23.36	0.193
PI522183A	Glyine soja	CN	48.48	127.97	424.23	11.12	0.026
PI522184	Glyine soja	CN	48.48	127.97	111.91	21.02	0.188
PI532449	Glyine soja	CN	45.00	135.00	313.32	20.79	0.066
PI532452A	Glyine soja	CN	41.00	126.00	300.13	6.16	0.021
PI549032	Glyine soja	CN	52.98	127.36	130.66	3.35	0.026
PI549035A	Glyine soja	CN	40.55	124.07	209.38	7.98	0.038
PI549036	Glyine soja	CN	40.55	124.07	319.13	15.03	0.047
PI549039	Glyine soja	CN	40.55	124.07	328.41	3.89	0.012

*: CN: China; JP: Japan; SK: South Korea; RU: Russia

Table S2. SNP effect and heritability estimate for the significant SNPs related to plant specialized metabolic pathway.

**Significant SNP**	**Chromosome**	**Position**	**LOD score**	**SNP effect**	**% R-squared value**	**h^2 (%)**
**ss715585948**	Gm03	38591888	3.80	-0.31	10.58	35
**ss715603454**	Gm09	30262482	3.80	0.06	4.22	
**ss715603455**	Gm09	30191235	3.80	0.05	3.32	
**ss715603462**	Gm09	30393285	3.80	-0.06	3.96	
**ss715603471**	Gm09	30725658	3.80	-0.06	3.86	
**ss715615975**	Gm13	37748250	3.81	0.22	12.59	
**ss715620269**	Gm15	11003656	3.81	-0.20	11.42	
**ss715636844**	Gm20	15930392	3.79	-0.13	4.16	

Locations of the significant SNPs shown in base pairs, bp; standardized SNP effects, and the percentage (%) of the total phenotypic variation (glyceollin induction) explained by significant SNPs (% R-squared values) on different chromosomes. LOD scores represent the chromosome-wide significant level from 3.79 to 3.82. Heritability (h^2) of glyceollin induction was calculated with all SNPs.

Table S3. Annotation of candidate genes other than genes within cluster 1 and 2.

Genes	Annotation
Glyma.20G052000, Glyma.20G052400	UDP-Glycosyltransferase superfamily protein
Glyma.20G057500	UDP-glucosyl transferase 85A2
Glyma.20G058000	hydroxy methylglutaryl CoA reductase 1 (Mevalonate pathway I; isoprenoid biosynthetic process; sterol biosynthetic process; coumarin biosynthetic process; oxidoreductase activity)
Glyma.20G053900	*cytochrome P450*, family 71, subfamily B, polypeptide 34 (Oxidoreductase activity)
Glyma.20G065000, Glyma.20G065100	*cytochrome p450* 79a2
Glyma.13G272500	*bZIP* transcription factor
Glyma.09G125400, Glyma.13G272700, Glyma.13G273500, Glyma.13G277600, Glyma.15G134700, Glyma.20G064600	*RING/U-box* superfamily protein, *RING/FYVE/PHD* zinc finger superfamily protein
Glyma.03G176600, Glyma.09G129100, Glyma.09G127100 Glyma.15G139000, Glyma.15G135600	*WRKY* family transcription factor family protein
Glyma.09G113000, Glyma.09G113100, Glyma.15G134100	*myb* domain
Glyma.03G173100, Glyma.03G173200,	zinc fingers superfamily protein

Glyma.03G173300, Glyma.09G128700, ***Glyma.09G115100,*** ***Glyma.09G107400*** ***Glyma.13G274600, Glyma.20G059300***	
Glyma.13G274300, Glyma.13G279900, Glyma.13G280000	*NAC* transcription factors
Glyma.09G115900, Glyma.09G117400, Glyma.09G123200, ***Glyma.13G277100,*** ***Glyma.20G053900, Glyma.20G065000, Glyma.20G065100***	*cytochrome P450* enzyme family
Glyma.20G065000, Glyma.20G065100	*cytochrome p450* 79a2 (oxidoreductase activity; Linamarin biosynthesis**)**
Glyma.09G108800	Zinc finger, *RING-type*; Transcription factor jumonji/aspartyl beta-hydroxylase
Glyma.09G109500, Glyma.09G122500	Terpenoid cyclases family protein, terpene synthase 03
Glyma.03G176300	Glutathione S-transferase family protein (glucosinolate biosynthetic process)
Glyma.09G126500 Glyma.09G126900 ***Glyma.15G139200***	phenylpropanoid metabolic process

Table S4. Gene of interest with enzyme class and associated metabolic domain for chromosome 9.

Gene Cluster	Gene Name	Enzyme Class	Signature or Tailoring?	Metabolic Domain
**Cluster 1**	*Glyma.09G127200*<br>*Glyma.09G127300*	glycosyltransferase	tailoring	Phenylpropanoid Derivatives; Specialized Metabolism
**Cluster 2**	*Glyma.09G127700*<br>*Glyma.09G128200*<br>*Glyma.09G128300*<br>*Glyma.09G128400*	glycosyltransferase	tailoring	Phenylpropanoid Derivatives; Specialized Metabolism

Table S5. Annotation of candidate genes of gene clusters 1 and 2.

Genes	Annotation
Glyma.09G127200 **(cluster 1)**	*UGT88A1* (AT3G16520.3); isoflavone 7-O-glucosyltransferase (2.4.1.170); metabolic process (GO:0008152); hexosyltransferase activity (GO:0016758), glucosyl/glucuronosyl transferases (PTHR11926); UDP-glucoronosyl and UDP-glucosyl transferase (PF00201)
Glyma.09G127300 **(cluster 1)**	*UGT88A1* (AT3G16520.3); isoflavone 7-O-glucosyltransferase (2.4.1.170); metabolic process (GO:0008152); hexosyltransferase activity (GO:0016758); glucosyl/glucuronosyl transferases (PTHR11926); UDP-glucoronosyl and UDP-glucosyl transferase (PF00201); glycosyltransferases (K08237)
Glyma.09G127700 **(cluster 2)**	*UGT88A1* (AT3G16520.3); isoflavone 7-O-glucosyltransferase (2.4.1.170); metabolic process (GO:0008152); hexosyltransferase activity (GO:0016758), glucosyl/glucuronosyl transferases (PTHR11926); UDP-glucoronosyl and UDP-glucosyl transferase (PF00201)
Glyma.09G128300 **(cluster 2)**	*UGT88A1* (AT3G16520.3); isoflavone 7-O-glucosyltransferase (2.4.1.170); metabolic process (GO:0008152); hexosyltransferase activity (GO:0016758), glucosyl/glucuronosyl transferases (PTHR11926); UDP-glucoronosyl and UDP-glucosyl transferase (PF00201)
Glyma.09G128200 **(cluster 2)**	UDP-glucosyl transferase 88A1 (AT3G16520.3); metabolic process (GO:0008152); intracellular membrane-bounded organelle (GO:0043231); transferase activity; transferring hexosyl groups (GO:0016758); quercetin 3-O-glucosyltransferase activity (GO:0080043); quercetin 7-O-glucosyltransferase activity (PWY-2345); biochanin A conjugates interconversion (PWY-2861); isoflavone 7-O-glucosyltransferase (GN7V-57685)
Glyma.09G128400 **(cluster 2)**	*UGT88A1* (AT3G16520.3), metabolic process (GO:0008152), hexosyltransferase activity (GO:0016758), glucosyl/glucuronosyl transferases (PTHR11926), and UDP-glucoronosyl and UDP-glucosyl transferase (PF00201)
